# The role of extracellular vesicles in mediating progression, metastasis and potential treatment of hepatocellular carcinoma

**DOI:** 10.18632/oncotarget.12465

**Published:** 2016-10-04

**Authors:** Naibin Yang, Shanshan Li, Guoxiang Li, Shengguo Zhang, Xinyue Tang, Shunlan Ni, Xiaomin Jian, Cunlai Xu, Jiayin Zhu, Mingqin Lu

**Affiliations:** ^1^ Department of Infection and Liver Diseases, Ningbo First Hospital, Ningbo, China; ^2^ Department of Infection and Liver Diseases, The First Affiliated Hospital of Wenzhou Medical University, Institute of Liver Research, Wenzhou Medical University, Wenzhou, China; ^3^ Department of The First Clinical Medical, Wenzhou Medical University, Wenzhou, China; ^4^ Department of Respiration, Lishui People's Hospital of Wenzhou Medical University, Lishui, China; ^5^ Laboratory Animal Center, Wenzhou Medical University, Wenzhou, China

**Keywords:** extracellular vesicles, exosomes, miRNA, hepatocellular carcinoma, MSCs

## Abstract

Hepatocellular carcinoma (HCC) is a major cause of cancer-related death worldwide. As vectors for intercellular information exchange, the potential role of extracellular vesicles (EVs) in HCC formation, progression and therapy has been widely investigated. In this review, we explore the current status of the researches in this field. Altogether there is undeniable evidence that EVs play a crucial role in HCC development, metastasis. Moreover, EVs have shown great potential as drug delivery systems (DDSs) for the treatment of HCC. Exosomal miRNAs derived from HCC cells can enhance transformed cell growth in recipient cells by modulating the expression of transforming growth factor-β activated kinase-1(TAK1) and downstream signaling molecules. Furthermore, vacuolar protein sortin 4 homolog A(VPS4A) and insulin-like growth factor(IGF)-1 regulate exosome-mediated miRNAs transfer. Immune cells- derived EVs containing integrin αMβ2 or CD147 may facilitate HCC metastasis. In addition, EVs-mediated shuttle of long non-coding RNAs (lncRNAs), specifically linc- VLDLR and linc-ROR promote chemoresistance of malignant cells. Heat shock proteins (HSPs)-harboring exosomes derived from HCC tumor cells increase the antitumor effect of natural killer (NK) cells, thus enhancing HCC immunotherapy. Indeed, inhibition of HCC tumor growth has been associated with tumor cell-derived exosomes (TEX)-pulsed dentritic cells (DCs). Exosomes are also essential in liver metastasis during colorectal carcinoma (CRC) and pancreatic ductal adenocarcinomas (PDAC). Therefore, as nucleic acid and drug delivery vehicles, EVs show a tremendous potential for effective treatment against HCC.

## INTRODUCTION

Hepatocellular carcinoma (HCC) is the sixth most common incident cancer worldwide and the third leading cause of cancer death annually [1, 2]. In China, HCC is one out of the four leading causes of cancer-related death [3]. The development of primary liver tumors, including HCC and cholangiocarcinoma are associated with hepatocyte damage. Large-scales studies have thoroughly described not only the pathogenesis of HCC development and metastasis [4-6] but also the multiple treatment options for HCC treatment, including surgical resection, orthotopic liver transplantation (OLT), transcatheter arterial chemoembolization (TACE), systemic or regional chemotherapy, and targeted immunotherapy [7, 8]. Recently, the emerging role of extracellular vesicles (EVs) in HCC progression and therapy attracted considerable attention.

Although EVs - including exosomes and microvesicles (MVs) - were previously considered as cellular debris, they are currently well-recognized vectors for intercellular exchange of information [9]. EVs mediate intercellular communication by transferring biologically and functionally active proteins and RNA across cells. Exosomes with 30-120 nm diameter are formed by release of intracellular multivesicular bodies, whereas MVs with a 120-1000 nm diameter, are formed by cell membrane shedding [10]. Many cells, including neurons [11], dendritic cells (DCs) [12], B cells, T cells, hepatocytes [13], stem cells [14], erythrocytes [15], mast cells, epithelial cells [16], tumor cells [17], along with some multicellular parasites [18-20], have the capacity to release exosomes. Exosomes can be usually found in biological fluids such as blood, urine and ascitic fluid [21-23] and they are usually detected using presence of CD63, tumor susceptibility gene(TSG)-101, alix and absence of endoplasmic reticulum marker Grp94 and calnexin, peroxisome marker protein (PMP)70, mitochondria marker COX IV [24-27]. Exosomes have been described as a means of communication between tumor cells [28]. Dysregulation in this cell-to-cell communication and undesirable cellular cross-talks are considered to contribute to cancer development and progression. A growing body of evidence already described that uptake of exosomes stuffed with proteins, mRNAs, miRNAs and lipids could deliver biological information that regulate the function of target cells [17, 29]. This mechanism may explain how exosomes mediate tumor progression and metastasis. Exosomes interact with their target cells mainly via fusion of membranes and transfer of exosomal contents, especially miRNAs.

HCC tumor cell-derived EVs have been reported to potentially contribute to local spread, intrahepatic metastases and multifocal growth of HCC [28]. EVs-mediated intercellular transfer of biologically active RNA and proteins might enable HCC cells to affect the tumor microenvironment, thereby causing HCC development and metastasis [30]. Experimental and clinical studies have elucidated the role of EVs in HCC development and metastasis, in order to be employed in future novel therapies against HCC including immunotherapy and chemotherapy, as biomarkers or as drug delivery systems (DDSs). Among these studies, human HCC cell lines Huh-7, HepG2, Hep3G, Hep3B, PLC/PRF/5, SMMC-7721, HKCI-8, FHCC-98, HKCI-C3, MHCC97L and mouse HCC cell lines including H22 were used to investigate the role of EVs in HCC cells *in vitro*.

Accumulating evidence indicate that EVs are involved in tumor progression, metastasis, and treatment failure, thus showing great potential as DDSs for the treatment of HCC. In the present article, current studies investigating the mechanism on the contribution of EVs to HCC development, progression, metastasis and treatment are reviewed.

## CONTENTS AND FUNCTIONS OF HCC-DERIVED EVS

### Components and tumorigenic mechanism of HCC-derived exosomes

To rapidly and efficiently extract exosomes secreted by tumor cells, different methods were explored [31-34]. Among them, sequential ultracentrifugation is the method of choice to isolate exosomes from culture supernatant of HCC cells in a consistent manner.

The RNA expression profile of exosomes derived from human HCC cell lines Hep3B and PLC/PRF/5 was investigated [28]. Interestingly, the exosomal was below 200 bases in size (mainly miRNAs) with a very low fraction of internal control genes, including 18S ribosomal RNA (rRNA), 28S rRNA, small nuclear RNA (snRNA) U6, small nucleolar RNA (snoRNA) U38B and snoRNA U43 [28]. The expression of 11 miRNAs (miR-584, miR-517c, miR-378, miR-520f, miR-142-5p, miR-451, miR-518d, miR-215, miR-376a, miR-133b and miR-367) was specifically detected in Hep3B-derived exosomes, indicating selective enrichment of a specific set of miRNAs in HCC-derived exosomes [28]. Similarly, a total of 20 miRNAs were detected exclusively in PLC/PRF/5-derived exosomes. There was a moderate correlation between the findings in both cell lines, indicating the existence of a sorting mechanism that guides HCC cells to secrete specific intracellular miRNAs into exosomes. The incubation of HCC cells with a neutral sphingomyelinase 2 (nSMase) inhibitor GW4869 resulted in an unchanged cellular expression and reduced exosomal expression of miR-16, indicating that the release of specific miRNAs from HCC cells into exosomes might occur *via* a ceramide dependent manner [28, 35]. Interestingly, some of these miRNAs (e.g. miR-451) were also found to preferentially enter exosomes in many other cell types [36].

**Figure 1:**
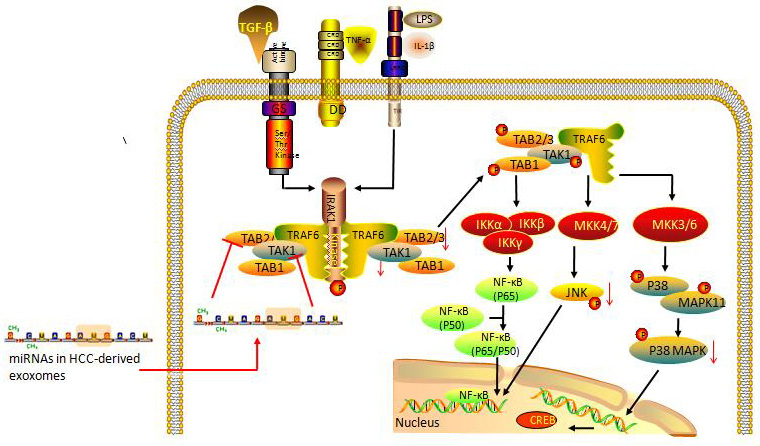
HCC-derived exosomal miRNAs may mediate tumor progression through modulating the TAK1-associated signaling pathway in recipient cells TAK1 is involved in the activation of signaling cascades mediated by IL-1, TNF-α, TGF-β in physiological conditions (black arrows). After uptake of HCC-derived exosomes by neighboring cells present in the tumor microenvironment, the given exosomal miRNAs can down-regulate the constitutive expression of TAK1, TAB 2/3 in recipient cells and restrain the phosphorylation of TAB1, TAB2/3 and TAK1. Activation of their three main downstream signaling pathways, including IKK α/β-NF-kB, MKK4/7-JNK-API and MKK3/6-p38/MAPK-CREB is then suppressed. The pathological unbalance finally causes failure of cellular homeostasis, leading to tumorigenesis in the liver and HCC tumor progression. (Abbreviation: TAB, TAK-1 binding protein; IKK, inhibitor of nuclear factor kappa-B kinase; MKK, MAPK kinase; CREB, cAMP response element binding protein).

HCC-derived exosomes mediated miRNA transfer is an important mechanism of environmental modulation of HCC growth and progress [28]. While being taken up and internalized, HCC-derived exosomes transfer their miRNAs contents into recipient cells to mediate transmission of functional transgenes and genetic modulation of cellular activities. The transfer of exosomal miRNAs regulates target gene expression, cell signaling, biological behavior and transformation of recipient cells. A combinatorial analysis on 108 potential genes identified that the transforming growth factor-β activated kinase-1 (TAK1) pathway might be a very likely candidate pathway targeted by these miRNAs [28]. TAK1 has been extensively associated with the activation of signaling cascades mediated by interleukin(IL)-1, tumor necrosis factor (TNF-α) and transforming growth factor(TGF)-β [37]. It is an upstream member of the mitogen-activated protein (3) kinase(MAP3K) family and an essential component of cellular homeostasis, intercellular communication and tumorigenesis in the liver. Loss or downregulation of TAK1 in hepatocytes is linked to HCC [38]. The modulation of TAK1 expression and associated signaling pathways in recipient cells could represent an important mechanism of exosomal miRNA mediated HCC tumor progression (Figure 1). HCC-derived exosomes can transfer their miRNA contents into recipient cells, inhibit the constitutive expression of TAK1 and downstream signaling associated with TAK1, and consequently lead to HCC development and metastasis. In this direction exosomes derived from Hep3B cells are able to both increase anchorage-independent growth of transformed cell and modestly reduce cell viability of recipient cells [28].

Some exosomal miRNAs and long non-coding RNAs (lncRNAs) are involved in HCC progression and treatment failure. For the first time, Li *et al*. reported that enhanced expression of miR-429 in liver tissue - caused by hypomethylation may be used as a prognosis factor in HCC patients [39]. The enrichment of miR-429 in HCC cells, especially in epithelial cell adhesion molecule (EPCAM)^+^ -tumor initiating cells (T-ICs) could lead to shedding of miR-429-harboring exosomes, thus facilitating tumor formation. These exosomes could shuttle and relocate miR-429 into their surrounded target cells, targeting Rb binding protein 4 (RBBP4) expression and further promoting the transcriptional activity of E2F1, finally upregulating the expression of POU class 5 homeobox 1(POU5F1) [39] in recipient cells. As a result, exosomes crucially contribute to hepatocyte self-renewal, tumorigenicity, malignant proliferation, chemoresistance and progression. Furthermore, exosomal transfer of the lncRNA regulator of reprogramming (linc-RoR) may contribute to failure of anticancer therapies and HCC development by increasing HCC resistance against adverse environmental conditions, such as hypoxia [40]. The levels of linc-RoR in normal hepatocytes are low [40] potentially inhibiting cell proliferation and tumorigenesis, in part, by specifically increasing stability of the c-myc mRNA [41]. High expression of linc-RoR in HCC decreases the expression of miR-145, a linc-RoR target, thereby increasing hypoxia inducible factor-1α (HIF-1α) and pyruvate dehydrogenase kinase isozyme 1 (PDK1) protein expression, overall improving mitochondrial function during hypoxia *via* the tricarboxylic acid cycle in recipient cells. Similarly, Takayuki and collaborators demonstrated that the most highly expressed lncRNA in HCC cell-derived EVs was TUC339. Suppression of TUC339 with short interfering RNA (siRNA) significantly reduced HCC cell proliferation and adhesion. Therefore, EVs-mediated transfer of lncRNA-TUC339 is a unique signaling mechanism to promote HCC growth and metastasis [42].

### HCC suppressors/promoters exert effects by exosome-mediated miRNAs shuttle

Based on the evidence that vacuolar protein sorting 4 homolog A (Vps4A) is frequently down-regulated in human HCC tissue and that Vps4A represses the colony formation, migration, growth and invasion of HCC cells *in vitro*, Vps4A was identified as a HCC suppressor [43]. Vps4A utilized exosomes as mediators to modulate secretion, uptake and final profiles of miRNAs in HCC cells [43]. In this study, Wei and colleagues compared SMMC-7721 cells transfected with either Vps4A (SMMC-Vps4A) or exosomes secreted by SMMC-Vps4A (SMMC-Vps4A-exo). They observed that Vps4A suppressed the bioactivity of exosomes *via* selectively packaging oncogenic miR-27b-3p and miR-92a-3p into exosomes and accumulating tumor-suppressive miR-193a-3p, miR-320a, and miR-132-3p in HCC cells. Moreover, they demonstrated that Vps4A decreased the recipient HCC cell response to exosomes *via* selective uptake of exosomal tumor-suppressive miR-122-5p, miR-33a-5p, miR-34a-5p, miR-193a-3p, miR-16-5p, and miR-29b-3p.

However, insulin-like growth factor-1 (IGF-1) is considered as a HCC promoter since it can override homeostasis and lead to tumor progression during the initial steps of HCC development [44]. Expression of tumor suppressor miR-122, a liver-specific anti-proliferative miRNA, is usually down-regulated in HCC cells compared with that in normal hepatocytes surrounding the tumor [45]. Transfer of exosomal miR-122 from healthy hepatocytes inhibits tumor progression. However, this method for the maintainance of homeostasis cannot be kept for a long time. T-ICs subsequently release IGF-1 to prevent miR-122 production in neighbouring normal hepatocytes and thereby curtail its intercellular transfer within exosomes, leading to low levels of the anti-proliferative miRNA in HCC cells. Thus eventually tumor progression and metastasis occurs [46].

## ROLE OF EVS IN HCC METASTASIS, CHEMORESISTANCE AND POTENTIAL IMMUNOTHERAPY

### Immune cells-derived EVs facilitate HCC metastasis

Immune cells such as immature myeloid cells, macrophages, and mast cells are considered the roots of metastasis of tumor cells [47, 48]. In a recent study [49] murine innate immune cells-derived microparticles (MPs) were co-cultured with H22 tumor cells, leading to tumor cell migration, invasion, attachment to the endothelium *in vitro* and metastasis *in vivo*. Indeed, MPs mediate the acquisition of a metastatic phenotype by HCC cells *via* the effective relay of integrin α_M_β_2_ (CD11b/CD18) from stimulated innate immune cells. These findings reveal that HCC tumor cells may usurp the phenotype of innate immune cells through MPs in order to metastasize. In patients with HCC, stromal and vascular invasions contribute to tumor progression. Kornek and colleagues indicated that MPs containing CD147 can be released by human T cells, stimulating the expression of matrix metalloproteinases (MMP) in fibroblasts and thus facilitating tumor invasion and metastasis [50].

### EVs-mediated delivery of special proteins promote HCC invasion and migration

The uptake of exosomes from invasive HCC cell lines can trigger the activation of phosphatidylinositol 3-kinase/protein kinase B (PI3K/AKT) and mitogen-activated protein kinase (MAPK) signaling pathways, which resulting in increased secretion of active MMP, enhanced migratory and invasive abilities of non-motile immortalized hepatocytes [24]. This may potentially lead to increased protrusive activity of HCC cells through the liver parenchyma during the process of metastasis. An *in vitro* study [51] demonstrated that up-regulation of annexin A2 (ANXA2) in HCC cells contributes to the expression of CD147, carried by their MVs. Moreover, highly expressed CD147 is responsible for the increased production of MMP-2 by fibroblasts in the liver stroma, thereby leading to HCC cells invasion and migration. Vasorin (VASN) is a transmembrane glycoprotein that plays an important role in vasculogenesis and tumor development [52]. Progression of HCC greatly depends on the communication between cancer cells and endothelial cells, which is mediated by tumor-derived exosomes. Exosomal transfer of VASN from HCC cells promotes mobility properties in hepatic endothelial cells, inducing cancer cell migration to the surrounding tissue [53](Table 1).

**Table 1:** Overview on the Contents and Functions of EVs Related with HCC

Molecules	EVs type	Donor cells(cell lines)	Recipient cells(cell lines)	Functions in HCC cells	References
Vasorin	exosomes	HCC Cells (HepG2)	Human umbilical Vein Endothelial Cells(HUVECs)	promote HCC cell migration	54
CD147	MPs	HCC Cells (SMMC-7721,FHCC-98)	Fibroblasts (HPF-1)	promote HCC cell migration and Invasion by up-regulation of MMP-2 in fibroblasts after stimulated by up-regulation of ANXA2 in donar cells	52
Integrin αMβ2	MPs	murine innate immune cells (splenic cells)	murine HCC cells (H22)	facilitate HCC metastasis in vivo and lead to tumor cell migration, invasion, attachment to the endothelium in vitro	50
linc-VLDLR	Evs	HCC Cells (HepG2)	HCC Cells (HepG2)	mediate acquired resistance to chemotherapy by enhancing expression of ABCG2 in HCC cell	56
lnc-TUC339	EVs	HCC cells (Hep3B, HepG2, PLC/PRF/5)	HCC cells (Hep3B, HepG2, PLC/PRF/5)	modulate HCC cell growth, proliferation and adhesion	43
linc-ROR	EVs	HCC cells (HepG2, Hep3B, PLC/PRF/5)	HCC cells (HepG2, Hep3B, PLC/PRF/5)	increase cell survival in recipient cells during hypoxia by modulating downstream miR-145–HIF1a-PDK1 signaling	42,58
miR-429	MVs	T-ICs	normal cells	promote liver T-ICs properties and facilitate HCC formation by targeting the RBBP4/E2F1/OCT4 axis in recipient cells	41
miR-122	exosomes	HCC Cells (Huh7)	HCC Cells (HepG2)	suppress HCC cell growth and proliferation	47
miR-122	exosomes	normal cells	T-ICs	inhibit tumor progression to maintain homeostasis, which is broken by IGF1(a HCC promoter) secretion in recipient cells to cause HCC progression	47
miR-142-3p	MVs	murine macrophages (Raw 264.7)	murine HCC Cells (Hepa1-6)	suppress HCC cell migration and invasion through down-regulation of RAC1(after propofol administration)	60
miR-27b-3p/ miR-92a-3p	exosomes	Human HCC cells (SMMC-7721, Hep3B)	Human HCC cells (SMMC-7721, Hep3B)	decrease HCC cell growth, migration and invasion ability after stimulated by Vps4A overexpression of donar cells	46

### EVs participate in inducing chemoresistance in HCC

HCC is a chemorefractory cancer and highly resistant to chemotherapy, limiting the effectiveness of anticancer agents [54]. Thus there is an urgent need for more efficient chemotherapeutic agents which overcome the mechanisms of chemoresistance in HCC cells.

EV-mediated transfer of linc-VLDLR is involved in HCC tumor cell response to chemotherapy by modulating cell-cell communication in the tumor microenvironment [55]. The essential role of VLDLR in mediating HCC chemoresistance was confirmed using linc-VLDLR which enhanced the expression of ATP-binding cassette sub-family G member 2(ABCG2) and increased chemotherapy- induced cell death, abrogated cell cycle progression and decreased cell viability [55]. Multidrug resistance is caused by overexpression of efflux transporters (such as P-glycoprotein), and exosomes can transfer P-glycoprotein intercellularly from multidrug-resistant donor cells to drug-sensitive recipient cells [56], thereby favoring multidrug resistance. During chemotherapeutic stress, TGF-β increases the expression of CD133^+^ cells and colony growth partly due to the selective enrichment and high expression of lincRNA-ROR (linc-ROR) within exosomes, overall inducing increased resistance of HCC cells to chemotherapy [57]. Considering the functional role of linc-ROR in TGF-β-dependent chemoresistance, the knockdown of ROR using exosomal siRNA delivery to enhance chemotherapy-induced apoptosis and cytotoxicity might be a potential therapy for the treatment of HCC.

However, MVs secreted by tumor-associated macrophages (TAMs) mediate effective HCC chemotherapy. In fact, propofol inhibits HCC cell invasiveness, viability and proliferation [58]. Macrophage activation and shuttling of miR-142-3p containing MVs from TAMs to HCC cells stimulated by propofol might be the underlying mechanism, leading to down-regulation of RAC1 - a target gene of miR-142-3p- expression, and thereby inhibiting tumor cell growth, migration and invasion [59].

### Exosomes mediating in enhanced immunotherapy of HCC

HCC is notoriously difficult to treat due to the unique immune tolerogenicity nature of the liver [60], however recent studies have reported that exosomes can counterbalance the hepatic immunosuppressive environment. The stress-induced extracellular heat shock proteins(HSPs) are known to confer tumor immunogenicity and induce natural killer (NK) cell antitumor responses [61]. Upon stimulation with chemotherapeutic drugs, HCC cells release HSP-bearing exosomes which enhance the cytolytic activity of NK cells and elicit efficient HSP-specific anti-HCC responses *in vitro* [62]. HCC-resistant or sensitive anticancer drugs differ in their ability to stimulate the production of HSP-bearing exosomes in HCC cells. Resistant anticancer drugs such as carboplatin and irinotecan hydrochloride generate more exosome-carried HSPs, which could up-regulate the expression of activating receptor CD69, NKG2D, NKp44, and down-regulate inhibitory receptor CD94 expression in NK cell, increasing granzyme B production and activating the NK cell cytotoxic response [62]. Thus, HSP-expressing exosomes can be potentially used as vehicles carrying therapeutic vaccines for HCC immunotherapy. A recent study using murine and human HCC cell lines demonstrated that DCs pulsed with HCC tumor cell-derived exosomes (TEX) can definitely elicit tumor suppression by improving tumor-specific immunity [63]. More importantly, intravenous injection of HCC TEX-pulsed DCs improves the tumor immune microenvironment, in terms of increased T cells and interferon(IFN)-γ levels and decreased IL-10 and TGF-β in tumor sites [63]. Additionally, several phase I clinical trials have used tumor-derived exosomes or exosome-pulsed DC as cancer vaccines [64, 65].

## ROLES OF EXOSOMES IN LIVER METASTASIS OF TUMOR

The chief cause of death in cancer patients is tumor metastasis [66] and the liver is one of the most commonly invaded sites. In most cases, liver metastases are derived from a colorectal or pancreatic tumor [67] [68] [69].

Several groups have demonstrated the pivotal role of exosomes in metastatic progression of colorectal carcinoma (CRC) [66]. The incubation of HepG2 cells with SW480 colorectal cancer cells-derived exosomes *in vitro* results in enhanced ability of HepG2 cell migration *via* activation of MAPK and extracellular signal regulated kinases (ERK)1/2 in recipient cells [70]. Besides, shuttling of exosomal microRNA (e.g. miR-21 [70] and miR-181a [71]) might mediate liver metastases of CRC by suppressing their target genes phosphatase and tensin homolog (PTEN), programmed cell death 4(PDCD4) or Wnt inhibitor factor(WIF)-1 in hepatocytes. Furthermore, it has been reported that HT-29 CRC cells are highly metastatic to the liver while Caco-2 CRC cells are poorly metastatic. A Chinese report [66] indicated that the administration of HT-29-derived exosomes to Caco-2-inoculated nude mice led to a pronounced enhancement of metastasis of Caco-2 cells to the liver. The underlying mechanism might be that HT-29-derived exosomes increase C-X-C chemokine receptor type 4 (CXCR4) expression in liver stromal cells, promoting a pro-inflammatory environment in the liver that favors metastasis.

**Figure 2:**
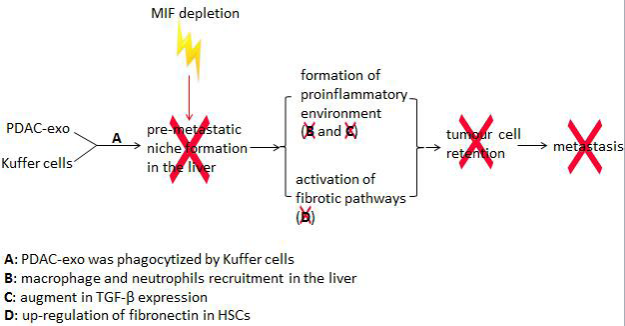
MIF is involved in PDACs-derived exosomes mediated pre-metastatic niche formation in the liver PDAC-derived exosomes fuse with Kupffer cells (KC) to prime the liver for metastasis by forming a pro-inflammatory environment and inducing the activation of fibrotic pathways, which favors the metastasis of pancreatic cancer cells to the liver. MIF is crucial in the pathogenic mechanism of pancreatic cancer-liver metastasis.

The liver is also the most common organ in metastases of pancreatic ductal adenocarcinomas (PDACs) [69]. Costa-Silva *et al*. clarified that PDACs-derived exosomes prime the liver for metastasis *via* pre-metastatic niche formation in the liver, in which highly expressed migration inhibitory factor (MIF) in PDAC-derived exosomes is very likely to be involved [72, 73] (Figure 2). Further studies are needed to show which exosomes components are involved in liver metastasis in different cancer cell types.

## ROLE OF EVS IN CHOLANGIOCYTES FORMATION

A recent study found that EVs secreted by the liver fluke *Opisthorchis viverrini* are implicated in cholangiocarcinoma formation via delivery of tumorigenic phenotype to cholangiocytes [74]. Authors showed that EVs internalization by cholangiocytes *in vitro* lead to cholangiocarcinoma formation *via* enhanced production of IL-6, cell proliferation, dysregulated expression of cancer-associated proteins (e.g. PAK-2, a kinase that mediates tumor cell invasion; ZO-2, a tight junction protein), and proteins involved in the proteasome complex [74]. Vaccines, which target tetraspanin 1, a key EV surface molecule, and subsequently interrupt the *O. viverrini* EV uptake process by cholangiocytes have shown great potential in the treatment of infection-derived cholangiocarcinoma.

## USE OF MESENCHYMAL STEM CELLS (MSCS) IN HCC TREATMENT

### MSC-derived EVs inhibit HCC development

Mesenchymal stem cells (MSCs) are multipotent cells with intrinsic properties of migration and tumor tropism. In recent years, a considerable number of studies have investigated the biological effect of MSCs on HCC tumor growth and progression [75-77]. Most studies demonstrated an antitumorigenic effect of MSCs in HCC growth and cell proliferation; however, several reports have postulated that MSCs could also be protumorigenic by promoting tumor vascularization, the tumor stem cell niche and thereby favoring tumor initiation [78-80]. Consistently, exosomes derived from MSCs induced the inhibition of HCC cell proliferation and growth both *in vitro* and *in vivo*, by exosomal delivery of selective proteins, mRNAs and miRNAs into HCC cells. In stem cell-based therapies, adipose- and bone marrow-derived MSCs are commonly used.

Sheung-Fat *et al*. conducted the first animal study using adipose-derived mesenchymal stem cells (AMSC)-derived exosomes for the treatment of HCC. The authors evaluated the effects of AMSCs-derived exosomes on N1S1 rat HCC cells inoculation-induced ectopic hepatoma *in vivo* [77]. Interestingly, AMSC-derived exosomes facilitated HCC suppression mainly by increasing intratumoral and circulating NK T-cells [81]. In their study, the exosome-treated rats harbored more intratumoral invariant CD8^+^ NK T-cells and circulating protective NK T-suppressing HCC growth. Considering that several miRNAs are associated with potential antitumor activity, AMSC exosomes were used for these miRNAs delivery [82, 83]. Since MiR-122 is a liver-specific anti-proliferative miRNA [84], miR-122-laden AMSC exosomes rendered HCC cells sensitive to chemotherapeutic agents both *in vitro* and *in vivo* [85], indicating that AMSC exosome might be novel carrier molecules for antitumor miRNAs in HCC treatment.

Similarly, MVs derived from bone marrow MSCs (BMSCs) inhibit cell cycle progression and induce apoptosis in HepG2 cells *in vitro*. Moreover, *in vivo* intra-tumor administration of BMSCs-derived MVs in tumors remarkably inhibited tumor growth [75]. The possible mechanism was not investigated. Although BMSCs could effectively inhibit HCC growth and progression via their MVs, homologous TEX-pulsed BMSCs possess stronger migratory capacity and exhibit more effective antitumor activities in HCC treatment than BMSCs alone, representing an innovative and alternative antitumor therapy. In the same direction, Ma [76] reported that murine homologous TEX-pulsed BMSCs could inhibit proliferation of H_22_ cells *in vitro*, as indicated by cell cycle arrest at G0/G1phase and significantly decreased PCNA protein expression in these cells. Previous studies [86-88] found that the antitumor activity of BMSCs may be significantly enhanced by cytokines such as IL-2 and IFN-γ. Indeed, BMSCs pulsed with TEX also exhibit enhanced migratory capacity, indicating that TEX could endow MSCs with greater migration ability and tumor tropism. BMSCs might uptake and present antigens, major histocompatibility complex (MHC) complexes and HSP of TEX, to induce effective antigen-specific cellular immune responses. This results in enhanced antitumor activity against HCC, very likely the underlying mechanism. Besides, human adult liver stem cells-derived MPs induce apoptosis and inhibit the proliferation of HepG2 cells *in vitro* and suppress growth of HCC xenografts in SCID mice by delivering antitumor miRNAs (miR451, miR223, miR24, miR31, miR214, and miR122), that down-regulate MDR1, MIF, ras-related protein 14(RAB14) and E2F-2 [89].

Altogether, these studies suggest that MVs derived from stem cells may inhibit HCC tumor growth and stimulate apoptosis in a variety of ways, including delivery of selected miRNAs, modulation of NK T-cell responses or antigen-specific T cellular immune responses. Regardless of the mechanisms, the complex antitumor capacity of MVs derived from MSCs or MSCs pulsed with homologous/autologous TEX in HCC treatment needs further investigation.

### AMSCs-derived EVs as miR-122 vehicle increase HCC chemosensitivity

Contrary to lincROR mediating TGF β-dependent chemoresistance, miR-122 promotes chemosensitivity of HCC cells. MiR-122 is a liver-specific anti-proliferative miRNA that can be transferred via exosomes between human hepatoma cells. The loss or down-regulation of miR-122 has been associated with HCC development and progression and is closely related to poor prognosis and metastasis of HCC [45, 90]. Moreover, high profiles of miR-122 render cancer cells sensitive to chemotherapeutic agents through down-regulation of miR-122-target genes expression in HCC cells, including cyclin G1 (CCNG1), a disintegrin and metalloprotease 10 (ADAM10), and IGF1-R. Moreover, transfection of donor cells with selective miRNA expression plasmids, high levels of these miRNAs are detected in their EVs. Lou showed that AMSC transfected with a miR-122 expression plasmid increase the therapeutic effect of chemotherapeutic agents such as 5-fluorouracil or sorafenib on 122-Exo-treated HCC cells. Thus, increased sensitivity of HCC cells to sorafenib by 122-Exo administration depends on exosome-mediated miR-122 transfer and down-regulation of miR-122-target genes is involved in the antitumor activity of sorafenib *in vivo* [85]. MSC is well suited for mass production of exosomes that are ideal for drug or miRNA delivery [91]. AMSC-derived exosome is a safe and effective vehicle for miR-122 delivery, and a key factor in miR-122-mediated chemotherapy sensitization. Furthermore, exosomes can be manufactured in culture by incorporating therapeutic miR-122 into exosome producing cells, thereby enabling personalized treatment [82]. Delivery of miR-122 *via* AMSC exosomes with use of chemotherapeutic agents results in enhancement of cell apoptosis and cell cycle arrest at G0/G1, what represents a promising strategy for HCC chemotherapy. In addition, exosomes from AMSCs have also been tested as an effective vehicle to package and deliver therapeutic siRNA [92] and active drugs such as paclitaxel [93]. Improvement in the methodology for AMSC culture and exosome purification will definitely increase the feasibility and safety of AMSC-derived exosome therapy in clinical treatment of HCC.

Similarly, the ability of intestinal epithelial cells Caco-2-derived MVs has been investigated as a vehicle for transfer of miR-168a to HCC cells. Caco-2 transfected by miR-168a expression plasmid secrete MVs containing these plant miRNAs. Thus, co-culture of these MVs with HepG2 might trigger transfer of specific miR-168a to human hepatocytes resulting in a 100-fold increase of the miR-168a levels and significant decreased expression of the miR-168a target protein [94].

## EVS AND DRUG DELIVERY

HCC is highly resistant to chemotherapy. Sorafenib, 5-fluorouracil and doxorubicin are currently being used for systemic or locoregional therapies against HCC but exhibit limited efficacy. Therefore, the discovery of new therapeutic targets and the development of novel clinical approaches to enhance HCC chemosensitivity are urgently needed. Therefore, the use of exosomes/EVs as nucleic acid (miRNA, lncRNA, siRNA, mRNA, DNA) and drug delivery vehicles has gained considerable interest due to their excellent biodistribution and biocompatibility.

## CONCLUSIONS

Altogether, these data suggest that EVs play multiple roles in mediating progression, metastasis and thus can be used as a potential therapy for the treatment of HCC. Further investigations are needed to shed more light on the role of EVs in HCC development and its application in the clinic.
